# Associations Between Maternal Meal Frequency Patterns During Pregnancy and Neonatal Anthropometric Outcomes: A Quantitative Cross-Sectional Study

**DOI:** 10.3390/nu17152437

**Published:** 2025-07-25

**Authors:** Oana Liliana Atomei, Petronela Vicoveanu, Camelia Oana Iațcu, Florina Ioana Gliga, Calin Coriolan Craciun, Monica Tarcea

**Affiliations:** 1 Doctoral School, “G.E. Palade” University of Medicine, Pharmacy, Science, and Technology of Targu Mures, 540139 Targu Mures, Romania; 2 Department of Biomedical Sciences, College of Medicine and Biological Science, “Stefan cel Mare” University of Suceava, 720229 Suceava, Romania; oana.iatcu@usm.ro; 3 Department of Medical-Surgical and Complementary Sciences, College of Medicine and Biological Science, “Stefan cel Mare” University of Suceava, 720229 Suceava, Romania; petronela.vicoveanu@usm.ro; 4 Obstetrics and Gynecology Department, Emergency County Clinical Hospital “St. Ioan cel Nou”, 720224 Suceava, Romania; 5 Department of Pathophysiology, “G.E. Palade” University of Medicine, Pharmacy, Science, and Technology of Targu Mures, 540139 Targu Mures, Romania; florina.gliga@umfst.ro; 6 Department of Surgery III, “G.E. Palade” University of Medicine, Pharmacy, Science, and Technology of Targu Mures, 540139 Targu Mures, Romania; calin.craciun@umfst.ro; 7 Surgical Clinic, Clinic County Hospital Mures, 540103 Targu Mures, Romania; 8 Department of Community Nutrition and Food Safety, “G.E. Palade” University of Medicine, Pharmacy, Science, and Technology of Targu Mures, 540142 Targu Mures, Romania; monica.tarcea@umfst.ro

**Keywords:** maternal nutrition, meal frequency, pregnancy, neonatal anthropometry, birth weight, socioeconomic status, fetal growth

## Abstract

**Background/Objectives**: Maternal dietary behaviors, including meal frequency patterns, may influence fetal growth. This study examined the associations between maternal meal frequency patterns during pregnancy—categorized as structured, moderately irregular, or highly irregular—and neonatal anthropometric outcomes, including weight, length, head, chest, and abdominal circumferences, and Apgar score. A secondary objective was to assess whether maternal education and household income modify these associations. **Methods**: This cross-sectional study included 1025 mother–newborn pairs from a socioeconomically diverse Romanian cohort. Maternal meal frequency patterns were classified based on self-reported weekly consumption. Neonatal anthropometric outcomes were obtained from medical records. Multivariable linear regression models, adjusted for maternal and neonatal characteristics, assessed the associations between meal frequency patterns and birth outcomes. Interaction terms evaluated effect modification by maternal education and income. **Results**: Structured maternal meal frequency patterns were associated with a slight but significant reduction in neonatal length compared to highly irregular patterns (β = −0.36 cm; 95% CI: −0.68 to −0.04; *p* = 0.02). A borderline inverse association with birth weight was also observed (β = −63.82 g; 95% CI: −128.87 to 1.23; *p* = 0.05). No significant differences were found for other anthropometric indicators or Apgar score. Maternal education modified the association between moderately irregular patterns and chest circumference (β = 0.15 cm; 95% CI: 0.04 to 0.25; *p* = 0.003), while household income modified the association with abdominal circumference (β = 0.14 cm; 95% CI: 0.02 to 0.26; *p* = 0.02). **Conclusions**: Maternal meal frequency patterns were modestly associated with neonatal length, while socioeconomic factors modified specific anthropometric outcomes. These findings highlight the importance of considering social context in prenatal nutritional recommendations.

## 1. Introduction

Maternal dietary patterns, including meal frequency, are a modifiable factor associated with fetal development and neonatal anthropometric outcomes, such as birth weight and length. Recent studies have shown that specific maternal dietary behaviors, including meal timing and composition, may influence neonatal growth indicators and perinatal outcomes [1,2]. While extensive literature has demonstrated the role of dietary quality and caloric adequacy in pregnancy [3,4,5], the potential associations between maternal meal frequency patterns and neonatal anthropometric outcomes have only recently begun to receive research attention [6,7,8].

Recent evidence suggests that irregular eating schedules—particularly erratic meal distribution or energy intake during late-night hours—may impair maternal metabolic homeostasis and nutrient transfer to the fetus [9,10,11]. Disruptions in maternal feeding patterns have been associated with altered hormonal rhythms, reduced melatonin secretion, and dysregulated placental function, and disrupted clock gene expression in animal and human studies [9,12,13], which collectively may affect fetal development and birth outcomes [14,15,16]. Recent studies indicate that structured and consistent meal timing, alongside meal frequency, may influence intrauterine growth by modulating maternal circadian and metabolic homeostasis [17,18]. Dysregulated circadian clock genes during pregnancy have been implicated in placental lipid metabolism disturbances and lower birth weight [14], suggesting a potential role for meal timing in prenatal development [17].

Central metabolic hormones—particularly insulin, leptin, and cortisol—exhibit circadian oscillations and are sensitive to meal timing [19,20]. During pregnancy, increased insulin and leptin levels support maternal adipose accretion and nutrient transfer to the fetus [7,21]. However, these hormones also undergo circadian-phase-dependent fluctuations that modulate placental transporter expression and nutrient sensing [22,23,24]. Cortisol, with its peak secretion in the early morning, regulates placental enzymatic activity and tissue differentiation. Disruption of these rhythms can impair nutrient allocation, with downstream effects on neonatal anthropometric patterns and gestational outcomes [25].

Structured meal patterns, characterized by regular consumption of three main meals and scheduled snacks, provide stable metabolic cues, improve glycemic control, and may enhance placental nutrient transfer [9,10,26]. In contrast, long fasting intervals and meal skipping, particularly breakfast omission or delayed eating, have been associated with suboptimal birth outcomes and increased maternal ketone production [17].

Although circadian nutrition research is advancing, empirical evidence linking meal timing and frequency with comprehensive neonatal anthropometric indicators remains limited. Recent reviews suggest that night-time eating during pregnancy is associated with adverse birth outcomes such as reduced weight and altered body composition [9,11,27], but the role of structured meal frequency remains underexplored. The relationship between maternal meal distribution patterns and neonatal anthropometric outcomes, especially birth weight, remains insufficiently explored, with few studies addressing multiple growth indicators simultaneously [28,29,30].

Research on maternal nutritional status has also shown that anthropometric indicators such as mid-upper arm circumference (MUAC), body mass index (BMI), and maternal head circumference correlate with neonatal size at birth [31,32,33]. Nevertheless, the temporal structure of dietary intake is seldom considered alongside these factors. Studies assessing adherence to dietary patterns like the Mediterranean or Dietary Approaches to Stop Hypertension (DASH) diet highlight improved birth weight and neonatal length [26,34,35], but do not isolate the role of eating schedule or structured intake. Advanced maternal age has been linked to lower nutrient intake and adverse neonatal parameters, such as smaller head circumference [36], reinforcing the need to contextualize feeding patterns within broader maternal characteristics.

These findings suggest that not only the quality and quantity of maternal nutrition but also meal frequency patterns may significantly influence fetal growth trajectories and neonatal anthropometric outcomes. Robust empirical data examining these associations remain limited. Well-designed observational studies that incorporate comprehensive assessments of maternal meal frequency and dietary structure remain important to inform evidence-based prenatal nutritional recommendations.

This study aimed to examine the associations between maternal meal frequency patterns during pregnancy, categorized as structured, moderately irregular, or highly irregular, and neonatal anthropometric outcomes at birth, including weight, length, head, chest, and abdominal circumferences, and Apgar score. We hypothesized that highly irregular meal frequency patterns, characterized by the absence of structured main meals, would be associated with suboptimal neonatal anthropometric indicators compared to more structured patterns. A secondary hypothesis posited that these associations may be modified by maternal socioeconomic status, specifically education level and household income.

## 2. Materials and Methods

### 2.1. Study Design, Population and Sample Size

This was a quantitative cross-sectional study conducted on a sample of 1025 mother–newborn pairs hospitalized at the “St. Ioan cel Nou” County Emergency Clinical Hospital in Suceava, Romania. The recruitment period spanned from July 2024 to May 2025. Recruitment was performed approximately every 4–5 days, in accordance with the average duration of postpartum hospitalization (typically 3 days after vaginal birth and 5–7 days after cesarean delivery, provided no complications occurred). During each recruitment visit, systematic review of medical records was conducted to identify eligible participants based on the predefined inclusion and exclusion criteria.

Inclusion criteria were women aged over 18 years, regardless of gestational age, who gave birth to live-born singleton neonates, voluntarily agreed to participate in the study, signed informed consent, and completed the questionnaire.

Exclusion criteria included adolescent mothers, multiple births, intrauterine fetal death, fetal macrosomia, maternal type 1 diabetes mellitus, and gestational diabetes. Also excluded were mothers whose newborns died during hospitalization, those who died prior to discharge, those who declined to sign informed consent, and those who refused to complete the questionnaire.

Both term (≥37 weeks gestation) and preterm (<37 weeks gestation) births were included in the study sample. Data on gestational age at birth was extracted from hospital medical records and included as an adjustment factor in our multivariable regression models.

Mothers identified as eligible were individually approached in their wards, informed about the study’s aims and procedures, and invited to participate. Those who agreed provided written informed consent prior to completing the questionnaire. Participants who declined were not included in the study.

**Figure 1:**
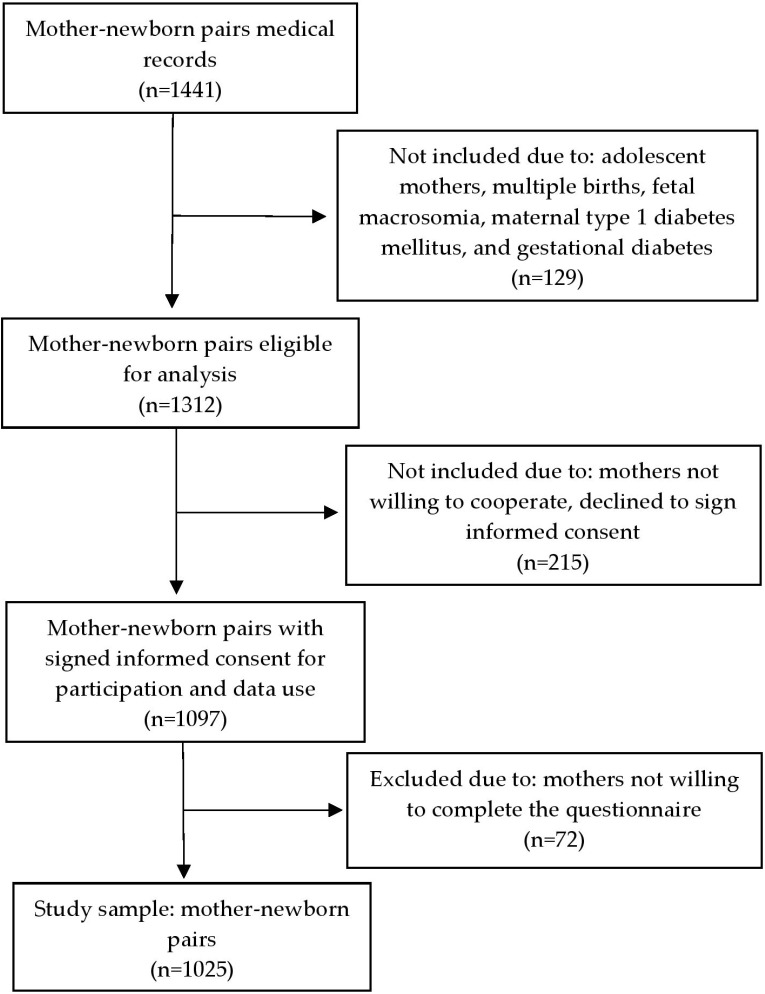
Flow chart showing selection of study participants.

Eligible participants (Figure 1) were selected after reviewing the medical records of the mothers and their newborns.

Maternal demographic information (age, marital status, place of residence), socioeconomic status (education level, income), obstetric profile (number of pregnancies, number of deliveries, gestational age at birth, type of delivery), anthropometric parameters (weight at admission, height), and clinical characteristics (hemoglobin, blood glucose, and blood pressure) were extracted from the obstetric medical records of women who gave birth.

Fasting glucose values ≥ 95 mg/dL (5.3 mmol/L) were used to identify gestational diabetes mellitus (GDM), based on established diagnostic thresholds [37]. Pregnancy-induced hypertension (PIH) was defined as systolic blood pressure ≥ 140 mmHg and/or diastolic pressure ≥ 90 mmHg, documented at hospital admission for delivery [38]. Hemoglobin values were obtained from routine complete blood count analyses performed at admission, with anemia defined as hemoglobin < 11 g/dL according to WHO guidelines [39].

These diagnostic criteria were applied to ensure consistency in the identification of exclusion factors—maternal type 1 diabetes mellitus, GDM, and fetal macrosomia—due to their recognized effects on dietary interventions and fetal growth trajectories. In the final study cohort, blood pressure and hemoglobin levels were retained as clinically relevant variables, as both anemia and PIH are known to influence placental function and fetal development. Their inclusion enabled adjustment for potential metabolic and vascular influences when analyzing the associations between maternal meal patterns and neonatal anthropometric outcomes.

Data regarding maternal meal frequency patterns were collected retrospectively within the first three days postpartum through a structured questionnaire that asked mothers to recall their usual meal frequencies throughout the entire pregnancy. No trimester-specific distinctions were made. Participants were invited to respond to the question: “How often have you had the following meals per week since you became pregnant?” Women reported the weekly frequency of seven eating occasions: morning snack, breakfast, lunch, afternoon snack, pre-dinner snack, dinner, and night meal. Response options ranged from 0 to 7 days per week. Another item assessed the intake of dietary supplements during pregnancy. This question about maternal meal patterns was used in the MoBa FFQ (Food Frequency Questionnaire), which is part of the Norwegian Mother and Child Cohort Study (MoBa) and publicly available at https://www.fhi.no/en/ch/studies/moba/for-forskere-artikler/questionnaires-from-moba/ (accessed on 1 December 2022).

Maternal pre-pregnancy BMI was derived from self-reported weight before conception and measured height, using the standard formula (kg/m^2^). Gestational weight gain was classified according to the recommendations outlined in the 2009 Institute of Medicine (IOM) guidelines [40]. Weight status was defined based on pre-pregnancy BMI as follows: underweight (BMI < 18.5), normal weight (18.5 ≤ BMI < 25), overweight (BMI ≥ 25), or obese (BMI ≥ 30) [41].

Household income was self-reported in predefined categories: 0 = no income, 1 = 1000–2000 RON, 2 = 2001–4000 RON, 3 = 4001–6000 RON, and 4 = >6000 RON per month. Income per capita was not calculated due to unavailable data on household size. Maternal education was recorded as the highest completed degree: lower secondary (≤8 years of schooling), high school, university degree, master’s, or doctorate. For statistical analyses, education was coded from 0 (lower secondary) to 4 (doctorate).

Information on neonatal outcomes—including sex, birth weight, length, head circumference, thoracic circumference, abdominal circumference, and Apgar score—was obtained from the electronic health records of the newborns. Apgar score was assessed at 1 and 5 min postpartum. This composite score ranges from 0 to 10 and includes heart rate, respiratory effort, muscle tone, reflex response, and skin coloration.

Including dietary supplement use allowed for a more accurate assessment of total maternal nutrient exposure, as supplements may independently influence fetal growth and could confound the relationship between maternal meal patterns and neonatal anthropometric outcomes.

### 2.2. Statistical Analysis

All statistical analyses were performed using MedCalc Statistical Software version 20.104 (MedCalc Software Ltd., Ostend, Belgium). Descriptive statistics summarized maternal, neonatal, and dietary characteristics. For continuous variables, distribution normality was assessed using the Shapiro-Wilk test, skewness, and kurtosis. Given the non-normal distribution of continuous outcomes, results are reported as medians with interquartile ranges (IQR), while categorical variables are presented as frequencies and percentages.

Bivariate associations between maternal meal frequency patterns and neonatal outcomes were first assessed using non-parametric tests. Specifically, the Kruskal–Wallis test compared median values of neonatal anthropometric outcomes (birth weight, length, head, chest, abdominal circumferences, and Apgar score) across three maternal meal frequency patterns: highly irregular, moderately irregular, and structured. When overall group differences were detected (*p* < 0.05), post-hoc pairwise comparisons were conducted using Dunn’s test.

For multivariable analysis, linear regression models examined the independent associations between maternal meal frequency patterns and continuous neonatal outcomes (birth weight, length, circumferences, and Apgar score). Dummy variables were created for the moderately irregular and structured patterns, with the highly irregular patterns serving as the reference category. Beta coefficients (βs) and 95% confidence intervals (CIs) estimated the adjusted mean differences in neonatal outcomes for each comparison group. All neonatal outcomes were analyzed as continuous variables, including Apgar score treated as a continuous approximation, to maintain consistency and comparability across models. No logistic regression analyses were performed.

Potential confounders were selected a priori based on literature and biological plausibility, including maternal socio-demographic factors (age, education, employment status, household income), obstetric characteristics (parity, gestational age at birth), maternal nutritional status (pre-pregnancy BMI, gestational weight gain), neonatal sex, anemia status, and supplement use. A series of progressively adjusted models were constructed: Model 1 (crude); Model 2 (adjusted for socio-demographics); Model 3 (Model 2 + parity and gestational age); Model 4 (Model 2 + nutritional status indicators); Model 5 (Model 2 + neonatal sex); and Model 6 (fully adjusted, adding anemia and supplement use). Results from the fully adjusted Model 6 are reported.

Interaction terms were introduced in the fully adjusted multivariable regression models to explore potential effect modification by maternal socioeconomic status. Specifically, interactions between maternal meal frequency patterns and either education level (coded 0–4) or household income (coded 0–4) were tested in relation to each neonatal outcome. To account for the reduced statistical power typically associated with interaction testing, a significance threshold of *p* < 0.10 was applied, in line with recommendations for exploratory epidemiological analyses. Where significant interactions were detected (*p* < 0.10), interaction coefficients (βs), 95% confidence intervals (CIs), and *p*-values were reported. No stratified subgroup analyses were conducted; interpretation was based on the direction and statistical significance of interaction terms within the fully adjusted models, which controlled for maternal age, employment status, household income, parity, gestational age, gestational weight gain, pre-pregnancy BMI, neonatal sex, anemia status, and dietary supplement use. Main effects were considered statistically significant at *p* < 0.05.

### 2.3. Ethical Considerations

The study received ethical approval from The Ethics Council of the “St. Ioan cel Nou” County Emergency Clinical Hospital in Suceava (reference no. 29/20.06.2024), The Ethics Committee for Scientific Research of the “George Emil Palade” University of Medicine, Pharmacy, Sciences, and Technology of Targu Mures (reference no. 3295/08.07.2024), and The Research Ethics Committee of the “Stefan cel Mare” University of Suceava (reference no. 216/20.06.2024). All participants were thoroughly informed about the study’s purpose and procedures and provided written informed consent. Participation was entirely voluntary, and all individuals retained the right to withdraw at any time, in accordance with the Declaration of Helsinki.

## 3. Results

### 3.1. Participant Flow, Maternal Characteristics, and Neonatal Anthropometric Outcomes

A total of 1441 mother–newborn medical records were screened for eligibility. After excluding 129 records due to adolescent mothers, multiple pregnancies, intrauterine fetal death, fetal macrosomia, and maternal diabetes (type 1 or gestational), 1312 pairs remained eligible for initial assessment. Among these, 215 mothers declined to sign informed consent or were not willing to cooperate. Of the 1097 mothers who consented to participate and data use, 72 did not complete the questionnaire and were subsequently excluded. The final analytical sample consisted of 1025 mother–newborn pairs (Figure 1).

Maternal sociodemographic indicators (Table 1) reflect a predominantly young, married, and economically vulnerable population. Nearly 88% of participating mothers were under the age of 35, and half resided in rural areas. Most women were employed (57.7%) and in stable relationships (92.2%). Regarding education, 38.2% had completed university studies, while 15.5% only had lower secondary education. For descriptive comparisons, income was dichotomized using 4000 RON/month as the cut-off, corresponding to the national net minimum wage in Romania (2025) [42]. Household income was low (≤4000 RON/month) in nearly two-thirds of families, underscoring a socioeconomically at-risk obstetric cohort.

From a clinical standpoint, anemia was prevalent in 31% of pregnant women, whereas pregnancy-induced hypertension was rare (0.6%).

Pregnancy and neonatal outcomes are detailed in Table 2. Vaginal delivery was the most common birth mode (52.1%), and most women were multiparous (63.4%). Term births accounted for 82.3% of cases, and neonatal gender was evenly distributed (49.6% male, 50.4% female).

Median maternal pre-pregnancy weight was 65 kg (IQR: 57–74), with a median gestational weight gain of 13 kg (9–16). The majority of mothers had a normal pre-pregnancy BMI (56.6%), while 25.2% were overweight and 13.2% were obese.

Neonatal anthropometric indicators showed a median birth weight of 3270 g (2900–3560), with 10.4% of newborns classified as low birth weight (<2500 g). Median birth length was 50 cm (48–51), and the median cranial, thoracic, and abdominal circumferences were 31 cm (31–33), 33 cm (32–34), and 35 cm (34–36), respectively. Apgar scores were high in most cases, with a median of 9 (8–9), suggesting generally favorable neonatal health at birth.

### 3.2. Maternal Meal Patterns and Frequency

#### 3.2.1. Weekly Meal Frequencies and Daily Consumption

Table 3 presents the weekly frequency distribution of each meal type. Lunch and dinner were the most consistently consumed meals, with median frequencies of 6 days/week, followed by breakfast (4 days/week). Snacking behaviors were more irregular, with lower medians and interquartile ranges. The lowest reported frequencies were for evening snacks and night meals. Only 30.9% of women reported daily breakfast consumption, and even fewer reported daily intake of snacks or night meals.

#### 3.2.2. Maternal Meal Frequency Patterns and Distribution During Pregnancy

Maternal meal frequency patterns were grouped into three broader categories based on daily meal structure and regularity: structured, moderately irregular, and highly irregular patterns (Table 4).

The structured group included women consuming three main meals per day, regardless of snack frequency, comprising 19.6% of the total sample. Specifically, 6.9% followed a fully regular pattern of three meals with multiple snacks (≥2 snacks/day), 7.2% consumed three meals with one snack daily, and 5.5% reported three meals without snacks.

The moderately irregular group encompassed participants who reported two main meals per day, accounting for 41.2% of the sample. This group included those consuming two main meals with one snack (23.9%), two main meals only (13.2%), and two main meals with multiple snacks (≥2/day) (4.1%).

The highly irregular group represented women with significantly reduced meal frequency, accounting for 39.2% of the total population. Within this group, 20.6% consumed only one main meal with snacks, while 18.6% had no main meals at all, relying exclusively on snacks throughout the day.

### 3.3. Associations Between Maternal Meal Frequency Patterns and Neonatal Anthropometric Outcomes

#### 3.3.1. Bivariate Analysis of Neonatal Outcomes by Maternal Meal Frequency Patterns

The distribution of neonatal anthropometric outcomes and Apgar scores across maternal meal frequency patterns is presented in Table 5. For birth weight, a statistically significant difference was observed between groups (*p* = 0.006). Post-hoc analysis revealed no significant pairwise differences (*p* > 0.05). For birth height, group differences were statistically significant (*p* < 0.001). Dunn’s test indicated significantly lower birth height in neonates from the structured patterns compared to the moderately irregular patterns (*p* < 0.001). Regarding head circumference, significant differences between groups were detected (*p* = 0.03), with a marginal association for lower head circumference in the structured group compared to the moderately irregular group (*p* = 0.05). Chest circumference also differed significantly across groups (*p* = 0.008), with lower values in the structured group compared to the moderately irregular group (*p* = 0.045). For abdominal circumference, the group comparison approached significance (*p* = 0.05), but post-hoc tests revealed no significant differences between groups (*p* > 0.05). Apgar score differed significantly between groups (*p* = 0.02). Post-hoc analysis showed a significantly lower Apgar score in neonates from the structured patterns compared to the moderately irregular group (*p* = 0.005). No significant differences were observed between the structured and highly irregular groups for any of the neonatal outcomes (all *p* > 0.05).

#### 3.3.2. Multivariable Regression Analysis of Maternal Meal Frequency and Neonatal Outcomes

Table 6 summarizes the results of the fully adjusted multivariable linear regression models (Model 6) assessing the association between maternal meal frequency patterns and neonatal anthropometric outcomes, controlling for maternal socio-demographic factors, obstetric characteristics, nutritional status, neonatal sex, anemia, and supplement use. Compared to the highly irregular patterns (reference category), neonates born to mothers with a structured meal pattern had significantly lower birth length (β = −0.36 cm; 95% CI: −0.68 to −0.04; *p* = 0.02). A marginal association was also observed for lower birth weight in the structured group relative to the highly irregular patterns (β = −63.82 g; 95% CI: −128.87 to 1.23; *p* = 0.05).

No significant differences were found for head circumference, chest circumference, abdominal circumference, or Apgar score between the structured and highly irregular patterns (all *p* > 0.05). Furthermore, no statistically significant associations were detected between the moderately irregular patterns and any neonatal outcome compared to the highly irregular group.

These associations remained consistent after adjusting for maternal socio-demographic characteristics, obstetric factors, nutritional status indicators, neonatal sex, anemia status, and supplement use (Model 6, fully adjusted).

#### 3.3.3. Interaction Analyses of Maternal Meal Frequency Patterns, Education Level, and Household Income in Relation to Neonatal Outcomes

Table 7 presents the interaction terms between maternal meal frequency patterns and maternal education level or household income in relation to neonatal anthropometric outcomes and Apgar score, based on the fully adjusted regression models. A statistically significant interaction was observed between the moderately irregular meal patterns and maternal education level for chest circumference (β = 0.09 cm; 95% CI: 0.03, 0.15; *p* = 0.003), indicating that the association between meal frequency and chest circumference varied by maternal education. Additionally, a significant interaction was identified between the moderately irregular meal patterns and household income for abdominal circumference (β = 0.17 cm; 95% CI: 0.03, 0.31; *p* = 0.02). A potential interaction trend was also observed between moderately irregular meal patterns and household income in relation to head circumference (β = −0.20, 95% CI: −0.43 to 0.02; *p* = 0.08). Although this did not meet the conventional threshold for statistical significance, the result may indicate a marginal association that warrants further investigation in future research. No statistically significant interactions were detected for other neonatal outcomes, nor for the structured meal patterns in relation to maternal education level or household income (all *p* > 0.10). These findings are based on fully adjusted models controlling for maternal socio-demographics, obstetric factors, nutritional status indicators, neonatal sex, anemia status, and supplement use.

## 4. Discussion

This cross-sectional study assessed the associations between maternal meal frequency patterns during pregnancy and neonatal anthropometric outcomes in a socioeconomically diverse Romanian cohort of 1025 mother–newborn pairs. Nearly 40% of women reported highly irregular meal frequency patterns, defined by the absence of structured main meals and reliance on snacks, reflecting a substantial prevalence of suboptimal eating behaviors in this population.

The categorization of maternal meal frequency into “structured” (three main meals per day), “moderately irregular” (two main meals), and “highly irregular” (≤one or no main meals, mostly snacks) was based on both clinical guidelines and empirical research evidence. International dietary guidelines consistently recommend that pregnant women consume three nutritionally balanced main meals daily, supplemented by healthy snacks, to meet increased caloric demands and to prevent prolonged fasting states that can destabilize maternal glucose metabolism and compromise fetal growth [40,43]. Romanian national guidelines echo these recommendations, encouraging small, frequent meals during pregnancy to support optimal maternal-fetal outcomes [44]. Supporting evidence from cohort studies strengthens this framework: women consuming all three main meals had significantly lower risk of preterm delivery in a population-based study of over 65,000 pregnancies [45], and increased risk of low birth weight and prematurity among women consuming only two meals per day [46], but irregular patterns—characterized by snacks and reduced meal structure—are associated with higher postprandial glucose and shorter gestational duration [47]. A recent review also underlined that consuming ≥3 meals per day reduces risks of low birth weight, anemia, and preterm birth [11]. Thus, our three-tiered classification aligns with both metabolic physiology and evidence-based public health guidance, offering a biologically and clinically meaningful framework for evaluating maternal meal frequency during pregnancy.

The primary finding was that adherence to a structured meal frequency pattern, characterized by the regular consumption of three main meals daily (with or without snacks), was independently associated with a slight but statistically significant reduction in neonatal birth length compared to highly irregular patterns (β = −0.36 cm; 95% CI: −0.68 to −0.04; *p* = 0.02). A borderline inverse association was also observed for birth weight (β = −63.82 g; 95% CI: −128.87 to 1.23; *p* = 0.05), though the small effect size and marginal significance require cautious interpretation. No significant differences were found for other neonatal outcomes, including head, chest, and abdominal circumferences or Apgar score (all *p* > 0.05).

Although structured maternal meal patterns were associated with a small but statistically significant reduction in neonatal length, no similar associations were observed for birth weight or head circumference. This apparent dissociation may be explained by the differential physiological regulation of fetal growth parameters. Linear skeletal growth is influenced by the insulin-like growth factor 1 (IGF-1) axis [48], which is responsive to maternal metabolic rhythms and may be modulated by meal timing and frequency [49]. It has been proposed that structured eating may stabilize maternal glucose metabolism but reduce the amplitude of postprandial anabolic responses [50], possibly attenuating fetal longitudinal growth compared to irregular patterns with higher glycemic variability.

In contrast, head circumference is typically preserved even under suboptimal nutritional conditions due to fetal brain-sparing mechanisms [51], which prioritize cranial development over somatic growth. Birth weight, as a composite outcome reflecting both lean mass and fat deposition, is subject to broader physiological influences that may dilute the effects of maternal meal structure alone [52]. These findings underscore the importance of evaluating individual anthropometric parameters separately, as they may respond differently to maternal dietary behaviors.

Our results partially support the study hypothesis by showing that maternal meal frequency patterns influence specific neonatal anthropometric parameters, particularly linear growth, while not significantly impacting overall size, proportionality, or immediate postnatal adaptation.

Interaction analyses revealed that maternal socioeconomic status modified these associations. A significant positive interaction was observed between moderately irregular meal patterns and maternal education in relation to neonatal chest circumference (β = 0.15 cm; 95% CI: 0.04 to 0.25; *p* = 0.003), suggesting that higher educational attainment may mitigate potential adverse effects of irregular meal frequency on fetal thoracic development. Similarly, household income moderated the association between meal patterns and neonatal abdominal circumference (β = 0.14 cm; 95% CI: 0.02 to 0.26; *p* = 0.02), emphasizing the role of socioeconomic resources in shaping fetal growth trajectories.

While the relationship between maternal meal frequency and neonatal anthropometry remains understudied, existing research links meal regularity and avoidance of meal skipping to improved birth outcomes, such as reduced risk of low birth weight and preterm birth [11,45]. Salunkhe et al. reported that consuming four or more meals daily was associated with lower risk of low birth weight, while restricted meal frequencies increased risk up to 12-fold [46]. Englund-Ögge et al. similarly found that regular main meals reduced preterm birth risk independent of other dietary factors [45].

Contrary to these studies, our results identified a modest inverse association between structured meal frequency and neonatal length, with no significant differences for weight or head circumference. One explanation may be that structured eating coexists with factors such as caloric restriction, health-conscious dietary restraint, or socioeconomic limitations that attenuate fetal growth.

Experimental evidence suggests that frequent and structured meal consumption contributes to stabilizing maternal glycemia but may attenuate peaks in insulin-like growth factor-1 (IGF-1), a key regulator of fetal skeletal development [49]. In contrast, irregular meal frequency may increase glycemic variability and stimulate fetal anabolic signaling, which could explain the slightly greater neonatal length observed among infants born to mothers with highly irregular meal patterns in our study.

It is plausible that women with irregular meal frequency compensated through frequent snacking or increased caloric intake during non-standard eating occasions, potentially preventing fetal growth restriction. Previous research has shown that energy-dense or high-protein food consumption, especially in overweight or obese pregnancies, is associated with accelerated fetal growth and increased risk of macrosomia [8,53,54].

The socioeconomic context further influenced these associations [55]. Higher maternal education likely reflects improved nutritional literacy and dietary quality, while greater household income may facilitate access to food resources that buffer the effects of irregular eating on fetal growth.

The high prevalence of highly irregular meal frequency patterns—reported by nearly 40% of women—reflects the socioeconomic vulnerability of the cohort, with two-thirds of households reporting low income (≤4000 RON) and 31% of women experiencing anemia during pregnancy. These factors likely contribute to irregular meal patterns and fetal growth outcomes, highlighting the need to consider structural determinants alongside individual dietary behaviors.

Strengths of this study include the large, diverse sample, the detailed assessment of multiple neonatal anthropometric parameters, and the exploration of socioeconomic modifiers.

Limitations include the cross-sectional design, which precludes causal inference. Although adapted from validated instruments (e.g., MoBa FFQ), measurement error, especially regarding snacks or irregular episodes, cannot be excluded. The study did not collect data on overall dietary quality, macronutrient composition, total caloric intake, portion sizes, or meal timing, limiting the ability to isolate the effects of meal frequency from broader nutritional factors.

Exclusion of high-risk pregnancies, including those with gestational diabetes, pre-existing type 1 diabetes, or fetal macrosomia, may limit generalizability to lower-risk populations. It is well established that the relationship between maternal nutrition and fetal outcomes is more pronounced in high-risk pregnancies [56]. Therefore, by excluding these cases, our analysis may have underestimated the potential impact of meal frequency in populations where dietary management plays a more critical role in mitigating pregnancy-related complications.

Although the Apgar score was included as a neonatal outcome, its limited variability (most infants scored between 9 and 10) and ordinal nature may compromise the assumptions of linear regression. Therefore, the interpretation of associations involving this outcome should be cautious. Future studies should consider using ordinal or non-parametric methods more appropriate for such distributions.

Residual confounding by unmeasured factors such as physical activity, sleep, stress, or other socioeconomic variables cannot be ruled out. In addition, irregular eating patterns may have been influenced by early pregnancy symptoms such as nausea, vomiting, or reduced appetite, which were not assessed. The interaction analyses were exploratory, based on a liberal significance threshold (*p* < 0.10), and require confirmation in larger, prospective cohorts. Finally, although statistically significant, the magnitude of associations—particularly the reduction in neonatal length of approximately 0.36 cm—was small and unlikely to be of clinical relevance, suggesting that meal frequency patterns are only one of many interacting factors influencing fetal growth.

Although interaction terms were examined, stratified analyses by maternal education and income were not conducted due to limited subgroup size. This constraint may have reduced our ability to detect differential effects across socioeconomic strata.

The retrospective assessment of maternal meal frequency shortly after childbirth presents several methodological challenges. The accuracy of maternal recall may be limited, especially when reporting behaviors from early pregnancy. Social desirability bias may also influence responses, with participants more likely to report structured or recommended eating patterns. Meal frequency and dietary behaviors may vary across pregnancy trimesters, due to physiological changes such as nausea or appetite fluctuations. Since the questionnaire captured a general overview of the entire pregnancy rather than trimester-specific data, potential variations over time were not measured. This limits the precision of exposure assessment and may affect the interpretation of associations. Future studies using prospective, repeated dietary assessments during pregnancy could provide a more accurate picture of maternal eating patterns and their relationship with neonatal outcomes.

## 5. Conclusions

This study provides novel insights into the relationship between maternal meal frequency patterns and neonatal anthropometric outcomes within a socioeconomically diverse population. While structured meal patterns, defined by regular consumption of three main meals daily, were associated with a slight reduction in neonatal birth length, the overall effect size was small and unlikely to have clinical relevance. No significant associations were observed for other neonatal growth parameters, highlighting that meal timing behaviors, in isolation, exert only modest influence on fetal development.

The modifying role of socioeconomic factors—such as maternal education and household income—underscores the need to interpret prenatal dietary behaviors within their broader social and economic context. These findings emphasize that interventions targeting prenatal nutrition should integrate flexible, balanced eating patterns with efforts to address dietary quality, energy sufficiency, and socioeconomic barriers to health. Further research, particularly longitudinal and mechanistic studies, is warranted to clarify these associations and inform evidence-based public health strategies.

## Figures and Tables

**Table 1:** Characteristics of the study population (*n* = 1025).

Characteristics		*n* (%)
Sociodemographic		
Mother’s age (years)	18–34	903 (88.1)
	≥35	122 (11.9)
Place of residence	Rural	503 (49.1)
	Urban	522 (50.9)
Mother education	Low (≤8 years in school)	158 (15.5)
	Medium	475 (46.3)
	University degree	392 (38.2)
Marital status	Married	945 (92.2)
	Single	80 (7.8)
Employment status of the mother	Housewife	434 (42.3)
	Employed	591 (57.7)
Household income/month (RON)	≤4000 RON (low)	661 (64)
	>4000 RON (high)	364 (36)
Clinical		
Anemia	No	707 (69)
	Yes	318 (31)
Hypertension	No	1019 (99.4)
	Yes	6 (0.6)

**Table 2:** Pregnancy and neonatal outcome characteristics (*n* = 1025).

Characteristics		*n* (%)
Delivery mode	Vaginal	534 (52.1)
	Caesarean section	491 (47.9)
Parity	Primiparous	371 (36.6)
	Multiparous	654 (63.4)
Gestational week	Term	844 (82.3)
	Preterm (≤37 weeks’ gestation)	181 (17.7)
Prenatal weight (kg)		65.0 (57.0–74.0)
Maternal weight at admission (kg)		78.0 (70.0–87.0)
Gestational weight gain (kg)		13.0 (9.0–16.0)
Mother height (cm)		1.65 (1.60–1.69)
Pre-pregnancy BMI (kg/m^2^)		23.7 (21.3–27.0)
Pregestational BMI (kg/m^2^)	Underweight (<18.5)	52 (5.1)
	Normal weight (≥18.5 and <25.0)	580 (56.6)
	Overweight (≥25.0 and <30.0)	258 (25.2)
	Obese (≥30.0)	135 (13.2)
Sex	Male	508 (49.6)
	Female	517 (50.4)
Birth weight (g)	<2500 g	107 (10.4)
	2500–4000 g	918 (89.6)
Birth weight (g)		3270 (2900–3560)
Birth height (cm)		50 (48–51)
Head circumference (cm)		31 (31–33)
Chest circumference (cm)		33 (32–34)
Abdominal circumference (cm)		35 (34–36)
Apgar score		9 (8–9)

**Table 3:** Frequency and distribution of weekly maternal meal intake by meal type (*n* = 1025).

Meal Type	Median (IQR), Days/Week	% Women’s Daily Consumption (7/7 Days)
Breakfast	4 (1–7)	30.9
Morning snack	3 (1–6)	19.7
Lunch	6 (3–7)	47.2
Afternoon snack	4 (1–6)	21.4
Dinner	6 (3–7)	44.6
Evening snack	2 (0–4)	12.8
Night meal	0 (0–2)	5.6

**Table 4:** Distribution of maternal meal frequency patterns during pregnancy (*n* = 1025). Categories are defined by the count of main meals and snacks consumed per day.

Meal Frequency Patterns	*n* (%)
**Structured patterns**	**201 (19.6)**
3 main meals and ≥2 snacks/day	71 (6.9)
3 main meals and 1 snack	74 (7.2)
3 main meals only	56 (5.5)
**Moderately irregular patterns**	**422 (41.2)**
2 main meals and ≥2 snacks	42 (4.1)
2 main meals and 1 snack	245 (23.9)
2 main meals only	135 (13.2)
**Highly irregular patterns**	**402 (39.2)**
1 main meal and snacks	211 (20.6)
No main meals, only snacks	191 (18.6)

**Table 5:** Bivariate analysis of neonatal outcomes by maternal meal frequency patterns.

Neonatal Outcomes	Maternal Meal Frequency Patterns			Kruskal–Wallis *p*-Value
	Structured (*n* = 201)	Moderately Irregular (*n* = 422)	Highly Irregular (*n* = 402)	
Birth weight (g)	3150 (2765–3452.5)	3300 (2950–3600)	3280 (2900–3590)	**0.006**
Birth height (cm)	49 (48–50)	50 (49–51)	50 (48–51)	**<0.001**
Head circumference (cm)	35 (34–35)	35 (34–36)	35 (34–36)	**0.03**
Chest circumference (cm)	33 (31–34)	33 (32–34)	33 (32–34)	**0.008**
Abdominal circumference (cm)	31 (29–32)	32 (30–33)	31 (30–33)	0.05
Apgar score	8 (8–9)	8 (8–9)	8 (8–9)	**0.02**

Data presented as median (interquartile range, IQR). Significant results (*p* < 0.05) from Kruskal–Wallis test are bolded. Post-hoc Dunn’s comparisons (*p*-values): Birth weight: structured vs. moderately irregular (*p* = 0.08); structured vs. highly irregular (*p* = 0.42). Birth height: structured vs. moderately irregular (*p* < 0.001); structured vs. highly irregular (*p* = 0.90). Head circumference: structured vs. moderately irregular (*p* = 0.05); structured vs. highly irregular (*p* = 0.99). Chest circumference: structured vs. moderately irregular (*p* = 0.04); structured vs. highly irregular (*p* = 0.70). Apgar score: structured vs. moderately irregular (*p* = 0.005); structured vs. highly irregular (*p* = 0.06).

**Table 6:** Fully adjusted multivariable linear regression of neonatal outcomes according to maternal meal frequency patterns (highly irregular patterns as reference category; Model 6 adjusted for maternal age, education, employment status, household income, parity, gestational age at delivery, pre-pregnancy BMI, gestational weight gain, neonatal sex, maternal anemia, and supplement use).

Neonatal Outcomes	β (95% CI) Moderately Irregular vs. Highly Irregular	*p*-Value	β (95% CI) Structured vs. Highly Irregular	*p*-Value
Birth weight (g)	−16.47 (−67.44, 34.50)	0.52	−63.82 (−128.87, 1.23)	0.05
Birth height (cm)	0.12 (−0.12, 0.36)	0.32	−0.36 (−0.68, −0.04)	**0.02**
Head circumference (cm)	0.04 (−0.14, 0.21)	0.69	−0.05 (−0.28, 0.18)	0.66
Chest circumference (cm)	0.01 (−0.21, 0.24)	0.89	−0.20 (−0.49, 0.09)	0.18
Abdominal circumference (cm)	−0.09 (−0.35, 0.17)	0.50	−0.07 (−0.39, 0.26)	0.32
Apgar score	0.06 (−0.04, 0.17)	0.26	0.08 (−0.07, 0.23)	0.30

β: Regression coefficient; CI: Confidence Interval; *p*-values derived from multivariable linear regression. A *p*-value < 0.05 was considered statistically significant. Bold indicates statistically significant results (*p* < 0.05).

**Table 7:** Interaction terms between maternal meal frequency patterns and maternal education level or household income in relation to neonatal outcomes (fully adjusted models).

Neonatal Outcomes	Interaction Term (Meal Patterns × Education/Income)							
	Structured Patterns × Education Level		Structured Patterns × Household Income		Moderately Irregular Patterns × Education Level		Moderately Irregular Patterns × Household Income	
	β (95% CI)	*p*-Value	β (95% CI)	*p*-Value	β (95% CI)	*p*-Value	β (95% CI)	*p*-Value
Birth weight (g)	−9.08 (−74.42, 56.27)	0.78	−2.58 (−34.03, 28.88)	0.87	−32.86 (−89.27, 23.54)	0.25	17.24 (−6.51, 40.99)	0.15
Birth height (cm)	0.002 (−0.320, 0.324)	0.98	0.015 (−0.140, 0.169)	0.85	−0.15 (−0.42, 0.11)	0.25	0.05 (−0.06, 0.16)	0.37
Head circumference (cm)	−0.20 (−0.43, 0.02)	**0.08**	−0.03 (−0.14, 0.08)	0.56	−0.09 (−0.29, 0.09)	0.33	0.05 (−0.02, 0.13)	0.18
Chest circumference (cm)	−0.01 (−0.30, 0.28)	0.93	0.02 (−0.11, 0.16)	0.71	0.15 (0.04, 0.25)	**0.003**	0.06 (−0.17, 0.31)	0.59
Abdominal circumference (cm)	0.11 (−0.21, 0.44)	0.50	0.07 (−0.09, 0.22)	0.39	0.05 (−0.23, 0.35)	0.69	0.14 (0.02, 0.26)	**0.02**
Apgar score	−0.02 (−0.17, 0.11)	0.69	0.01 (−0.05, 0.09)	0.60	0.02 (−0.09, 0.14)	0.66	0.03 (−0.01, 0.08)	0.18

β = regression coefficient; CI = confidence interval; significant interaction terms are in bold (*p* < 0.10). Fully adjusted for maternal age, education, employment status, household income, parity, gestational age at delivery, pre-pregnancy BMI, gestational weight gain, neonatal sex, anemia status, and prenatal supplement use.

## Data Availability

The original contributions presented in this study are included in the article. Further inquiries can be directed to the corresponding author.

## Abbreviations

The following abbreviations are used in this manuscript:

MoBa	Norwegian Mother and Child Cohort Study
FFQ	Food Frequency Questionnaire
MUAC	Mid-upper arm circumference
BMI	Body mass index
DASH	Dietary Approaches to Stop Hypertension
GDM	Gestational diabetes mellitus
PIH	Pregnancy-induced hypertension
